# Profiles of gifted students through the eyes of their teachers: identifying students at risk for underachievement

**DOI:** 10.3389/frcha.2026.1678145

**Published:** 2026-08-31

**Authors:** Fae Aimée van der Weijden, Lisette Hornstra, Evelyn H. Kroesbergen

**Affiliations:** 1 Research Institute of Child Development and Education, University of Amsterdam, Amsterdam, Netherlands; 2 Department of Education, Universiteit Utrecht, Utrecht, Netherlands; 3 Behavioural Science Institute, Radboud Universiteit, Nijmegen, Netherlands

**Keywords:** achievement, person-centered approach, profiles of gifted learners, stability, teacher observation, gifted, underachievement

## Abstract

**Introduction:**

Not all gifted students realize their full potential and many gifted students are underachieving. To examine whether gifted primary school students at risk for underachievement can be identified by their teachers early on, the current study aimed to examine (1) whether different profiles can be distinguished among gifted students based on teacher ratings of observable characteristics (classroom behavior, task commitment, educational performance, teacherchild relationship quality, and peer relations), (2) to what extent the identified profiles and students' profile membership are stable over time, and (3) to what extent profile membership is associated with achievement (growth).

**Methods:**

Participants were two cohorts of Grade 3 students from primary schools in all districts of the Netherlands achieving above the 90th percentile on a cognitive ability test (*N* = 1,303; M_age_ = 8,44 years), who were assessed again in Grade 6.

**Results:**

Latent Profile Analyses revealed four profiles of gifted students in Grade 3, which mostly represented general quantitative differences in teacher ratings: above average, average independent and low conflict, average, and below average. Similar profiles were found in Grade 6, having similar sizes as in Grade 3. Latent Transition Analyses showed that half of the students changed profile between Grades 3 and 6. Profile membership in Grade 3 was related to achievement in Grades 3 and 6 (reading fluency, reading comprehension, and math) and, to a small extent, to achievement growth between Grades 3 and 6.

**Discussion:**

This study suggests that quantitatively distinct groups of gifted students can be distinguished based on teacher ratings of students' characteristics and that students' cognitive and non-cognitive characteristics, as observed by their teacher, could help to identify students at risk for underachievement.

## 1 Introduction

Previous research has shown that 16% to 50% of gifted students in primary school are underachieving (1, 2). These students perform lower than expected based on their IQ level (3). As underachievement grows, children's self-concept and task importance declines (4). Over the last decades, researchers have investigated why some gifted students demonstrate excellent achievement, while others develop towards trajectories of underachievement (5, 6). In order to explain these differences between gifted students, researchers have suggested that distinct types, or profiles, of gifted students can be identified that differ from each other in cognitive, behavioral, social-emotional and motivational characteristics and subsequently in their educational needs (7, 8). Such profiles have gained widespread popularity in educational practice in different countries (9–11). However, empirical support for the existence of different profiles of gifted students is mostly lacking.

This study aims to fill this gap by investigating whether different profiles among gifted students can indeed be distinguished based on their cognitive, behavioral, social-emotional and motivational characteristics, as observed by their teachers. In addition, it is examined whether students remain in the same profile over time, and whether these profiles explain differences in achievement growth during the last years of primary education. By identifying which combinations (i.e., profiles) of cognitive, social-emotional, behavioral, and motivational factors can be distinguished among gifted students and by assessing their predictive value for achievement outcomes, both favorable and unfavorable constellations of student characteristics can be identified, which may help to identify students at risk for underachievement. By adopting a person-centered approach i.e., identifying patterns of student characteristics, this study provided insights into the heterogeneity of gifted students, and added to a better understanding of the complex interplay between multiple factors that are associated with achievement (growth) in gifted students. Although previous research has found support for heterogeneity among gifted students (6, 19), this study is among the first to examine this heterogeneity through teacher perceptions. Thereby, this study may contribute to insights into how gifted students at risk for underachievement can be identified early on by their teachers.

Intellectual giftedness can be defined as having high intellectual abilities (6). These abilities *allow* individuals to be successful across all academic domains, but do not necessarily *lead to* outstanding performance (4, 6, 12). Whether the cognitive potential of a gifted student indeed develops into outstanding performance, depends on a large variety of cognitive, behavioral, social-emotional, and motivational student characteristics (6). Although some researchers consider student characteristics, such as motivation or creativity, to be an integral part of the definition of giftedness (13, 14), we align with the view of giftedness as a developmental process (15) in which these factors are considered to be facilitating factors that help gifted students to develop their potential into outstanding performance as they grow older. Accordingly, giftedness refers to the possession of exceptional abilities or aptitudes that place an individual among the top 10% of their age group (16). An IQ test may be used to assess whether individuals demonstrate above-average abilities or aptitudes, indicating their potential for outstanding performance.

The view that the gifted form a homogeneous group has dominated the field for a long time (17, 18). However, there is great variation between gifted students (19) and not all gifted students develop their abilities into outstanding performance (6, 12). For example, in Australia, Germany, New Zealand and the USA, the percentage of underachieving gifted students in primary and secondary school ranges from 9% to 28% (2). There has been a considerable amount of research on factors that are associated with gifted students’ underachievement in school (6). In their review, Raoof and colleagues (6) describe that internal factors (e.g., self-concept, emotional well-being, self-regulation), as well as external factors (e.g., attitudes towards the teacher, peer relations, social acceptance) are associated with underachievement of gifted students. Findings emphasize that not only cognitive factors are associated with underachievement in gifted students, but also non-cognitive and environmental factors. Researchers have suggested that these factors interact with each other and that only some combinations of factors lead to outstanding performance, while other combinations lead to less favorable achievement outcomes (14, 16, 20).

To take into account the complex interplay of different student characteristics, researchers have increasingly used person-centered approaches (21–23). Whereas traditional variable-centered approaches focus on effects of separate variables, person-centered approaches enable researchers to identify prevalent patterns of student characteristics, called ‘profiles’ (24, 25). By identifying specific groups of gifted students (i.e., profiles) with unique combinations of cognitive, social-emotional, behavioral, and motivational factors and by assessing their predictive value for achievement outcomes, both favorable and unfavorable constellations of student characteristics can be identified. This provides insights into possible indicators of students at risk for underachievement. An advantage of this approach is that researchers can gain insight into the heterogeneity of gifted students and a better understanding of the complex interplay between multiple factors that are associated with achievement and achievement growth.

Qualitative studies have suggested that distinct profiles of gifted students can be identified. For example, Betts and Neihart (7, 8) distinguished six profiles of gifted students based on observations and interviews: the successful, the creative, the underground, the at-risk, twice/multi-exceptional, and autonomous learner. These profiles differed from each other in students’ cognitive, creative, social-emotional, behavioral, and motivational characteristics. For example, the *autonomous learner* profile was characterized by self-confidence, intrinsic motivation, independence, and high social skills, whereas the *at-risk* profile was characterized by inconsistent work quality, high levels of creativity, poor self-concept, and rejection. Such profiles have gained widespread popularity among educators, who have come to understand profiles as stable dispositions of gifted students and have used them to provide better guidance for their students (9–11).

Over the last decades, also some relatively small-scale quantitative studies have examined whether profiles can be distinguished among gifted students, based on specific combinations of student characteristics, namely psychological characteristics (26–34) cognitive characteristics (22, 35, 36), motivational characteristics (5, 37–39), or a range of characteristics (40). Results of these studies have suggested that different types of gifted students can be identified. Most studies have based their profiles on only a limited range of student characteristics.

Several questions regarding profiles of gifted students remain unanswered. Firstly, it is unknown whether and which different types of gifted students can be identified in a large-scale empirical study using a broader set of student characteristics. Secondly, to enable teachers to identify different types of gifted students and tailor their support accordingly, it is important to examine whether such profiles can be distinguished based on characteristics as observed by teachers. Thirdly, despite the emphasis on giftedness as a ‘process of talent development’ (15), most of previous studies have been predominantly cross-sectional, failing to account for developmental changes in these characteristics or in achievement growth trajectories.

A longitudinal design is needed to gain insight into whether profiles are stable dispositions of (gifted) students or rather temporary states. In addition, examining the association between these profiles and achievement growth may help to identify profiles that put gifted students at risk for underachievement. It could be that some profiles show favorable achievement outcomes initially, but little progress in achievement over time, indicating a distinct risk for underachievement.

The present study is the first to use longitudinal large-scale data to investigate profiles among gifted students in upper elementary school based on a set of observable teacher-perceived student characteristics, and their relation to achievement outcomes. We investigated the following research questions:

1. Which profiles of gifted students can be distinguished based on characteristics that are observable by the teacher?
2. To what extent are the constellations of the profiles and students’ profile membership stable over time?
3. To what extent is profile membership associated with achievement and achievement growth?

This study included a variety of factors that are observable by teachers: internal factors such as classroom behavior, task commitment and educational performance, and external factors including the teacher-child relationship quality and peer relations. Following attachment theory, three aspects of the teacher-child relationship were distinguished: closeness, conflict, and dependence (41, 42). Closeness is the warmth, trust, and open communication between teachers and children. Conflict is the disharmony and resistance in the teacher-child relationship. Dependency refers to teachers’ concern about children's overreliance on the support of the teacher. Based on prior research (6, 43), we expected that the aforementioned factors are related to gifted students’ achievement. By using assessments at two waves in Grades 3 and 6, we investigated the stability of profile membership and whether different profiles in Grade 3 are associated with different rates of achievement growth in the next three years.

We expected to distinguish different profiles of student characteristics, based on previous profile research among gifted students (5, 22, 37, 38) and on research on differences among gifted students (12, 19, 44). However, we had no specific expectations regarding the constellation of the profiles, as the currently used set of student characteristics has not been investigated earlier. With respect to profile stability over time, we expected to find changes in the constellation of profiles and profile size as students grow older, based on previous profile research among gifted students (37, 39, 45, 46). In other words, we expected students to make transitions from one profile to another. As the constellation of the profiles at both waves is unknown, no hypotheses about the proportion of students who will transition could be formulated. As previous research among general student populations found both positive and negative changes over time in several cognitive and non-cognitive characteristics (35, 45–48), we expected transitions from favorable to unfavorable profiles and vice versa. Lastly, we expected profiles characterized by high-quality teacher-child relationships and classroom behavior, a high number of peer relations, and/or high task commitment to be associated with higher achievement growth whereas profiles characterized by low levels of these characteristics were expected to be associated with lower achievement growth (6, 44, 49–51).

## 2 Materials and methods

### 2.1 Sample

**Figure 1:**
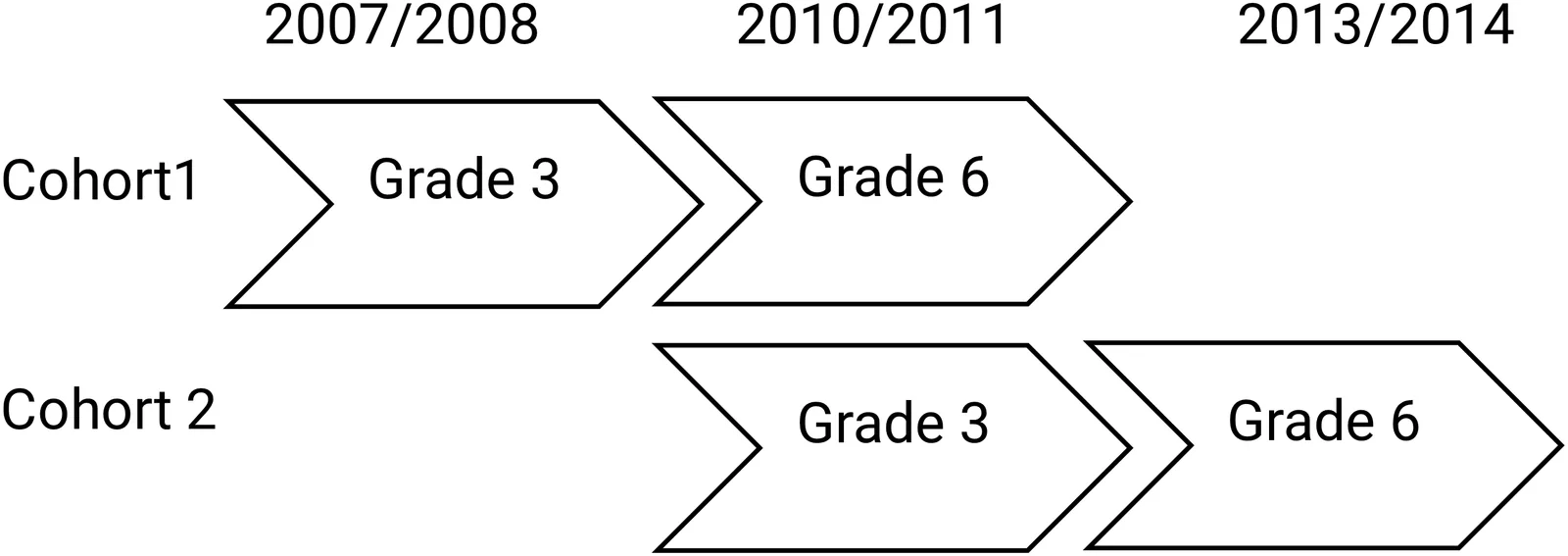
Overview of the Dutch COOL^5−18^ data.

For this study, we used data from the COOL^5−18^ project, a large-scale longitudinal study in primary and secondary education in the Netherlands, publicly available in the repository of Data Archiving and Networked Services (DANS (52);. The project included two cohorts of primary school students (*N_cohort1_* = 6,535; *N_cohort2_* = 6,008) in 778 classes in 444 schools in the Netherlands: the first measurement of the first cohort took place in 2007 and the first measurement of the second cohort in 2010. We used data from two cohorts of children that were measured three years apart. Both cohorts were assessed in Grade 3 and Grade 6 of primary school. For a visualization of the data collection, see Figure 1. The full sample of the COOL^5−18^ study consisted of 12,543 students from 444 primary schools and their 778 teachers. The sample of the COOL^5−18^ study was found to be representative for the Dutch population of primary school children (52).

The present study included those students who performed in the top 10% on a cognitive ability test (see Instruments) and who were therefore considered gifted students (16). This resulted in a sample of 1,303 gifted students from 338 schools and their 505 teachers. Children were between 6 and 10 years old at the first measurement wave (Grade 3). Schools were located in all of the twelve districts in the Netherlands, 1 to 19% per district (national sample: 3 to 17% per district). One fifth (22%) were rural schools and four fifths (78%) urban schools (national sample: 21% and 79%). The number of gifted children included in the sample of the present study ranged from 1 to 23 per school. Descriptive statistics of participants’ sex, nationality, socio-economic status, age, and scores on the cognitive ability test are displayed in Table 1. There was a relatively small number of children with parents with low educational levels (9%) and children with a migrant background (8%), compared to the overall average of the nationally representative COOL^5−18^ sample (25.5% and 16.1%, respectively).

**Table 1:** Descriptive statistics of Sex, nationality, SES, NSCCT, and Age.

Variable	Subgroup	n	%
Sex	Boys	627	47.60
	Girls	680	51.40
	Unkown	13	1.00
Nationality	Dutch	1135	85.95
	Migrant background	105	8.09
	Unkown	79	5.96
SES^1^	Low educational level	112	8.58
	Average educational level	506	38.42
	High educational level	623	47.04
	Unkown	79	5.96

Variable	M	SD	Minimum	Maximum
Age (Wave 1)	8.44	0.40	6.95	10.24
Cognitive ability (NSCCT^2^)	77.45	2.56	75.00	99.17

Values are pooled based on multiple imputed datasets (see Missing Data Handling).

1 Socio-economic status was indicated by the highest educational level obtained by one of the student's parents.

2 Non-Scholastic Cognitive Ability Test (see Instruments).

In Dutch primary education, gifted children are typically taught in regular mixed-ability classes. Facilities provided to gifted students in the classroom and outside of the classroom are usually dependent on whether the teacher recognizes the potential of a student and can accurately identify their educational needs (53). Typical adaptations within the classroom can include enrichment and acceleration. At the time of data collection, around a quarter of the schools in the Netherlands also provided facilities outside the classroom: participation in a so called ‘plus class’ (53). Here, children with above average cognitive abilities take challenging lessons for multiple hours per week.

### 2.2 Procedure

The data were collected in 2007–2014. At that time, formal ethical approval was not required for this type of research. Nevertheless, the institutions responsible for data collection adhered to all applicable institutional and national regulations, including relevant informed consent procedures*.* Passive consent was obtained from parents which meant that parents received a printed information letter explaining the purpose and procedures of the study. This letter was provided in four languages: Dutch, Turkish, Arabic and English. In this letter, it was explained that parents could reach out to the school, if they did not want their child to participate in the study, in which case, their child's data was not included in the study. After data collection, the anonymized data were published in an online repository (52). Children and schools had identifier codes and were not identifiable. In 2025, the Ethics Committee of Utrecht University approved the secondary use of the dataset for the present study (FERB-25-0345). The committee reviewed whether the study complied with current ethical guidelines and whether participants’ consent covered the intended use of the data.

### 2.3 Instruments

Data were collected between 2007 and 2014, see Figure 1. Children's cognitive ability was assessed in Grade 3, January–April. Their characteristics (cognitive, social-emotional, motivational, and behavioral) and school achievement were measured between January–April in both Grades 3 and 6.

#### 2.3.1 Cognitive ability

To identify the gifted student group, a cognitive ability test was specifically developed and psychometrically evaluated for the COOL^5−18^ study: the Non-Scholastic Cognitive Ability Test (NSCCT (54);. This test was administered in Grade 3 and consisted of 85 items divided into five subtests: (a) *Figure completion*, (b) *Exclusion*, (c) *Number series*, (d) *Categories*, and (e) *Similarities*. The first two subtests measured visual-spatial abilities, the third measured numerical abilities, and the last two subtests measured linguistic abilities. All subtests used multiple-choice questions with four answer possibilities. The test took one hour. For a more detailed description of the subtests see Driessen et al. (2015). Reliability and validity of the tests were evaluated as sufficient to good, according to the Dutch Committee on Tests and Testing (55). Cronbach's alpha coefficients for the five subtests ranged from .70 to .80, with a median reliability of .74, indicating sufficient reliability. Factor analyses revealed that these subtests form one general cognitive ability factor. Reliability of the total test was *α* = .91. Correlations between scores on the NSCCT and scores on standardized school achievement tests for math and reading comprehension ranged between *r* *=* .52 and *r* *=* .59, indicating sufficient convergent validity (54, 56).

#### 2.3.2 Child characteristics

Teachers from Grades 3 and 6 filled out a questionnaire on each student's cognitive, social-emotional, motivational, and behavioral characteristics (so each student was evaluated by two different teachers). This questionnaire consisted of two parts: (1) a teacher questionnaire of the PRIMA cohort study (57), and (2) a shortened version of the Student-Teacher Relationship Scale (58, 59). See Table 2 for a description, example items, and reliability of the scales. The total questionnaire consisted of 28 items. Answers were rated on 5-point Likert scales, with values ranging from 1 (definitely does not apply) to 5 (definitely applies). Originally, the questionnaire also contained negative scales, such as ‘teacher conflict’, and ‘teacher dependence’, but for the current study we renamed and recoded the negative scales, so that high scores indicate favorable scores for all scales.

**Table 2:** Descriptions, number of items, example items, and reliability of scales on the teacher questionnaire.

Scale	Number of items	Scale description	Example item	Cronbach's alpha
First part teacher questionnaire				
Educational performance	3	the discrepancy between student's ability and achievement (recoded)	"This student's performance lags behind his/her abilities”	.85
Classroom behavior	4	student's disruptive and rule-breaking behavior	“This student obeys the rules”	.81
Task commitment	3	student's conscientiousness and task persistence	“This students works precisely”	.82
Peer relations	3	student's contact and friendships with classmates	“This student has few friends in class”	.86
Second part teacher questionnaire				
Teacher independence	5	a developmentally inappropriate degree of overreliance and possessiveness of the child in the teacher-child relationship (recoded)	“This student asks for help when this is actually not needed”	.90
Lack of teacher conflict	5	the degree to which a teacher perceives teacher–student interactions as negative, discordant, unpredictable, and unpleasant (recoded)	“Dealing with this child is tiring”	.93
Teacher closeness	5	the degree of openness, warmth, and security in the relationship according to the teacher	“This student openly shares his/her feelings and experiences with me”	.87

#### 2.3.3 School achievement

In both Grades 3 and 6, standardized school achievement tests for reading fluency (*Drie-Minuten-Toets*), reading comprehension (*CITO Toets Begrijpend lezen*) and mathematics (*CITO Toets RekenenWiskunde*) were administered to assess students’ learning gains between Grades 3 and 6. The reading fluency test took three minutes. Both the mathematics test and reading comprehension test took 1 to 1.5 h, divided in two parts of 30–45 min each. For a more detailed description of the tests, see (52). For reading fluency, raw scores were obtained, representing the number of words read correctly in three minutes. For reading comprehension and math, continuous standardized ability scores (in Dutch: *vaardigheidsscores*) were obtained. Reliability and validity of the tests were evaluated as good, according to the Dutch Committee on Tests and Testing (60).

For the standardized school achievement tests of reading comprehension and math, two different versions were in use (reading comprehension: version 1997 and 2007, math: version 2002 and 2007). Some schools used either the old versions or the new versions in both grades, whereas other schools used different versions in Grades 3 and 6. For reading comprehension, scores on both versions were on the same scale, and thus comparable. For math, scores were not comparable. Therefore, scores of the version with the fewest observations (i.e., version 2002) were transformed, so that sample means and standard deviations of both versions were equivalent. To ensure that this could not affect the results of this study, a dummy variable for version was created, which was added as a covariate during analyses.

### 2.4 Missing data handling

The longitudinal data were subject to missing data and attrition. In Grade 3, 789 students (61%) fully participated and in Grade 6, 894 students (69%). In total, 11% of the data were missing (3 to 30% per instrument). Most cases with missing values on one scale of the teacher questionnaire or NSCCT also had missing values on all other scales of that measure (62 to 92%, depending on the grade). Moreover, many cases that had missing values on one standardized school achievement test also had missing values on (an)other standardized school achievement test(s) in that grade (58 to 75%, depending on the grade). Often, this was the case for whole classes or schools. Therefore, it was concluded that these missing values could be attributed to individual absence of students or to the fact that schools or teachers did not participate in certain measurements*.* This suggests that the missing values were Missing at Random (61). Generally, two approaches are recommended to handle missing data: maximum likelihood estimation and multiple imputation (60). With maximum likelihood estimation the same model is used to impute and analyze the data, while different models are used in multiple imputation. Multiple imputation therefore allows the researcher to use extra variables to predict missing values. We chose for multiple imputation, so that we could predict missing values in school achievement and child characteristics by non-missing data, as well as by variables outside the model: i.e., cohort membership, sex, socio-economic status, family composition, number of years living in the Netherlands, age, and having special needs, all obtained in Grade 3. By using five imputations and having 11% of missing values, we reached 98% efficiency, according to Rubin's formula (1 +  λ/*m*)^−1^, where λ is the rate of missing information and *m* is the number of imputations (60), in our study (1 + 0.11/5)^−1^ = 0.98. Multiple imputation was done in MPlus 7.0, taking into account the nested structure of students in classes (62).

### 2.5 Analytic strategy

We used two applications of the general latent-class model: latent profile analyses (LPA), and latent transition analysis (LTA). The goal of LPA is, as with other clustering techniques, to identify the smallest number of meaningful profiles through which the data can be described (24, 63, 64). LPA was preferred over the traditional clustering techniques (i.e., hierarchical or K-means clustering techniques) because of more rigorous statistical criteria to decide about the number of clusters and the information LPA provides about the accuracy of the classifications of students into profiles (24, 65, 66). This accuracy information is represented in the posterior probabilities for profile membership, which indicate the probabilities that students belong to a certain profile (66), and in the entropy measure, which assesses overall classification accuracy (67).

LTA is a longitudinal extension of LPA that is designed to model not only latent profile membership, but also transitions over time in latent profile membership (68–70). While LPA provides probabilities of cases belonging to a certain profile, LTA provides probabilities of cases remaining within a particular profile or changing to another profile.

#### 2.5.1 Preliminary analyses

Assumptions were evaluated prior to analyses. Normality was met for all variables. Outliers were defined as participants with a z-score that deviated more than 3.29 standard deviations from the mean (71). Outliers were only found on standardized school achievement tests. Outlier values were recoded to a value corresponding to a z-score of 3.29 standard deviations (71, 72). Multivariate outliers on the standardized school achievement tests and NSCCT, detected by their Mahalanobis distance, were deleted (*n* = 31) (71). The assumption of absence of multicollinearity was met. The intraclass correlation (ICCs) suggested that a substantial proportion of variance of the variables in this study (variables used in LPA/LTA and achievement variables) was situated at the class level (range ICC_class level_: 0.087–0.299). Therefore, the multilevel structure of the data was taken into account during analysis in MPlus ('type = complex’). The conditional independence assumption was met for all LPA and LTA models (66).

#### 2.5.2 Latent profile analyses

To identify profiles of gifted students, LPA (63, 64) was employed using MPlus 7.0 (62). In the LPAs, the scale scores for each of the student characteristics were modeled as indicators of a latent categorical variable. This latent variable represents the profiles to be distinguished in the data. Solutions with one to nine profiles were evaluated, for both Grades 3 and 6.

To determine the number of profiles, several criteria were evaluated. First, several fit statistics were considered: the Akaike Information Criterion (AIC) (73), the Bayesian Information Criterion (BIC) (74), and the Sample-Adjusted BIC (SA-BIC) (75). For these measures, lower values indicate a better balance between model fit and parsimony (70). Second, two tests were employed that compared a profile solution to a profile solution with one profile less: the Vuong-Lo-Mendell-Rubin Likelihood Ratio Test (VLMRT) (76), the Lo-Mendell-Rubin Adjusted Test (LMRA) (77). For both tests, significant *p*-values indicate that a specific model outperforms the same model with one profile less (78). Third, the size of the extracted classes was also considered to make sure each class was large enough to warrant interpretation. Profiles with few cases (e.g., <5% of the cases) were not considered theoretically meaningful. Fourth, we considered entropy values (ranging from 0 to 1), with a higher value reflecting more accurate classification (79). Fifth, the conceptual meaningfulness and interpretability of the solutions was considered.

#### 2.5.3 Latent transition analyses

To investigate the changes in profile membership over time, LTA (68, 69) was employed using MPlus 7.0 (62). Like the LPA, the LTA model included scale scores of the student characteristics as indicators for a latent categorical variable. However, in this model, the data from both measurement waves were included to indicate two latent categorical variables instead of one, representing the profiles at each measurement occasion. In addition, the current LTA models included coefficients that regressed profile membership at the second measurement occasion on profile membership at the first measurement occasion. These coefficients are multinomial logistic regression coefficients predicting profile membership at time t given profile membership at t-1. The value of these coefficients should be interpreted as the rate (or probability) of transitioning from one profile to another over time. The profile-specific means of the indicator variables depend on both profile and time. Full measurement invariance i.e., restricting the profile-specific means to be invariant across time, was tested as this facilitates interpretation. Outcomes are reported in the results section.

#### 2.5.4 Profile membership associations with achievement and achievement growth

To examine whether the identified profiles in Grade 3 and Grade 6 predicted achievement and growth in achievement, a three-step BCH method was used (80), using MPlus 7.4 (62). Although it is possible to use a one-step model and include distal outcomes (here: achievement) in the latent profile models, this could have led to undesirable shifts in the latent classes, because the latent classes would have been determined not only by their indicators but also by the distal outcomes (81). As an alternative, researchers (82, 83) have introduced three-step approaches in which they first estimate the latent profiles, then assign respondents to their most likely profile, and then estimate differences between these groups in distal outcomes, for example by a MANOVA analysis. However, this approach can lead to underestimations of the strength of the relationships between the latent profiles and distal outcomes, as the most likely profile is threated as an observed variable and classification error is not taken into account (84). By combining the three-step approach with a BCH bias correction method, these biases are prevented (85). As such, we used the three-step BCH method as described by Asparouhov and Muthén (80) to examine if (1) the profiles in Grade 3 were associated with achievement in Grade 3, (2) the profiles in Grade 6 were associated with achievement in Grade 6, and (3) the profiles in Grade 3 predicted achievement growth between Grades 3 and 6. To test for significance, we used Wald tests, and corrected for multiple testing, using the Šidák correction (86), resulting in a significance threshold of α = .0028. BCH weights were averaged over imputed datasets for each profile in each grade.

## 3 Results

The descriptive statistics of the measures of student characteristics and achievement tests are summarized in Table 3. Correlations between measures of student characteristics are displayed in Table 4.

**Table 3:** Descriptive statistics of the measures of student characteristics and achievement tests.

	Grade 3		Grade 6	
	*M*	*SD*	*M*	*SD*
Educational performance	2.77	0.82	2.87	0.86
Classroom behavior	3.91	0.77	3.94	0.80
Task commitment	3.83	0.81	3.91	0.86
Peer relations	3.75	0.78	3.78	0.81
Teacher independence	3.11	0.74	3.36	0.70
Lack of teacher conflict	3.43	0.71	3.52	0.71
Closeness to the teacher	3.72	0.63	3.64	0.69
Reading comprehension	38.59	13.14	69.70	16.63
Reading fluency	71.76	17.52	102.65	13.15
Math	85.47	11.88	121.26	9.87

Values are pooled based on multiple imputed datasets.

**Table 4:** Correlations between student characteristics.

Student characteristic	Pearson's r					
	1	2	3	4	5	6
Grade 3						
1 Educational performance	1.00					
2 Classroom behavior	.33*	1.00				
3 Task commitment	.52*	.55*	1.00			
4 Peer relations	.28*	.41*	.35*	1.00		
5 Teacher independence	.32*	.30*	.33*	.28*	1.00	
6 Lack of teacher conflict	.37*	.63*	.45*	.43*	.48*	1.00
7 Closeness to the teacher	.22*	.22*	.22*	.38*	.05	.31*
Grade 6						
1 Educational performance	1.00					
2 Classroom behavior	.36*	1.00				
3 Task commitment	.54*	.56*	1.00			
4 Peer relations	.25*	.31*	.31*	1.00		
5 Teacher independence	.28*	.29*	.32*	.24*	1.00	
6 Lack of teacher conflict	.35*	.64*	.44*	.31*	.45*	1.00
7 Closeness to the teacher	.26*	.32*	.34*	.33*	.02	.36*

*p < .01.

### 3.1 The prevalence of profiles among gifted students

Our first aim was to investigate which profiles of gifted students can be distinguished based on students’ cognitive, social-emotional, behavioral, and motivational characteristics. For Grades 3 and 6 gifted students, we evaluated LPA models with one to nine profiles. Model results are shown in Table 5. Different fit statistics pointed to different profile solutions. Although the VLMRT, LMRA, and entropy pointed towards a three-profile solution in both grades, the four-profile solution was considered the best representation of the sample in both grades, as AIC, BIC, and SA-BIC pointed towards solutions with more profiles, the smallest profile of the four-profile solution still contained more than 5% of the sample, and the entropy indicated clear classification. Finally, the constellation of the profiles of the four-profile solutions suggested that these solutions identified substantively meaningful and interpretable profiles. Profile mean scores on the student characteristics in Grades 3 and 6, resulting from the four-profile solutions, are shown in Table 6.

**Table 5:** Latent profile solutions for gifted students’ characteristics in Grade 3.

*k*	AIC	BIC	SA-BIC	VLMRT (*p*)	LMRA (*p*)	Entropy	Smallest profile (%)
Grade 3							
1	20950	21023	20979	-	-	1	100
2	19304	19418	19348	.001* −.06	.002* −.06	0.81	26.2
3	18453	18609	18514	.002* −.01*	.003* −.01*	0.95	7.0
**4**	**18135**	**18332**	**18212**	.002* −.18	.002* −.28	**0**.**85**	**7**.**1**
5	17785	18023	17877	.03* −.46	.03* −.46	0.88	3.1
6	17630	17910	17739	.04* −.13	.04* −.13	0.88	2.7
7	17692	18013	17816.	.09 −.48	.09 −.48	0.90	0.0
8	17472	17835	17612	.29 −.71	.29 −.71	0.86	0.6
9	17652	18057	17809	.22 −.72	.22 −.73	0.89	0.0
Grade 6							
1	21513	21586	21541	-	^­^-	1	100
2	19859	19974	19904	<.001*	<.001*	0.78	38.6
3	19284	19439	19344	<.001* −.02*	<.001* −.02*	0.83	7.2
**4**	**19043**	**19240**	**19120**	**.06 −.38**	**.06 −.40**	**0**.**82**	**5**.**4**
5	18892	19130	18984	.14 −.47	.14 −.48	0.87	1.3
6	18626	18906	18734	.03* −.17	.04* −.17	0.89	1.7
7	18508	18830	18633	.28 −.38	.28 −.38	0.87	1.2
8	18442	18805	18582	.06 −.29	.06 −.29	0.85	1.7
9	18674	19078	18831	.10 −.76	.10 −.76		0.0

For VLMRT and LMRA, this table contains ranges as parameters can vary between imputed datasets. *significant for *α* < .05.

The bold values represent the best profile solution.

**Table 6:** Classification of gifted students in Grades 3 and 6 profiles of student characteristics based on their most likely latent profile membership.

Student characteristic	Profile 1 (*n* = 526/581)	Profile 2 (*n* = 242/264)	Profile 3 (*n* = 461/406)	Profile 4 (*n* = 93/71)
Grade 3				
Educational performance	3.26	2.47	2.53	2.03
Classroom behavior	4.45	3.81	3.62	2.57
Task commitment	4.41	3.46	3.56	2.90
Peer relations	4.17	3.60	3.54	2.80
Teacher independence	3.49	3.18	2.80	2.28
Lack of teacher conflict	3.95	3.88	2.95	1.62
Teacher closeness	3.94	3.67	3.60	3.19
Grade 6				
Educational performance	3.35	2.51	2.59	1.94
Classroom behavior	4.53	3.78	3.47	2.54
Task commitment	4.53	3.49	3.54	2.81
Peer relations	4.14	3.55	3.59	2.95
Teacher independence	3.67	3.43	2.99	2.68
Lack of teacher conflict	3.99	3.66	2.95	1.65
Teacher closeness	3.93	3.49	3.45	2.91

Profile sizes in Grade 3/Grade 6 are separated by slash.

To examine whether the profiles in Grades 3 and 6 were comparable, full measurement invariance i.e., restricting the profile-specific means of indicator variables to be invariant across grades, was tested. Table 7 shows that for the model with means varying across grades, fit statistics were hardly better than for the model with grade-invariant means, leading to the decision to continue with the second model and to assume that profiles were equivalent in Grades 3 and 6.

**Table 7:** Model Fit statistics of a model With and without profile-specific means constrained across grades.

Model	AIC	BIC	SA-BIC	Entropy
*Model 1:* Profile-specific means vary across grades	37017	37458	37188	0.841
*Model 2:* Profile-specific means equal across grades	37105	37401	37220	0.840

**Figure 2:**
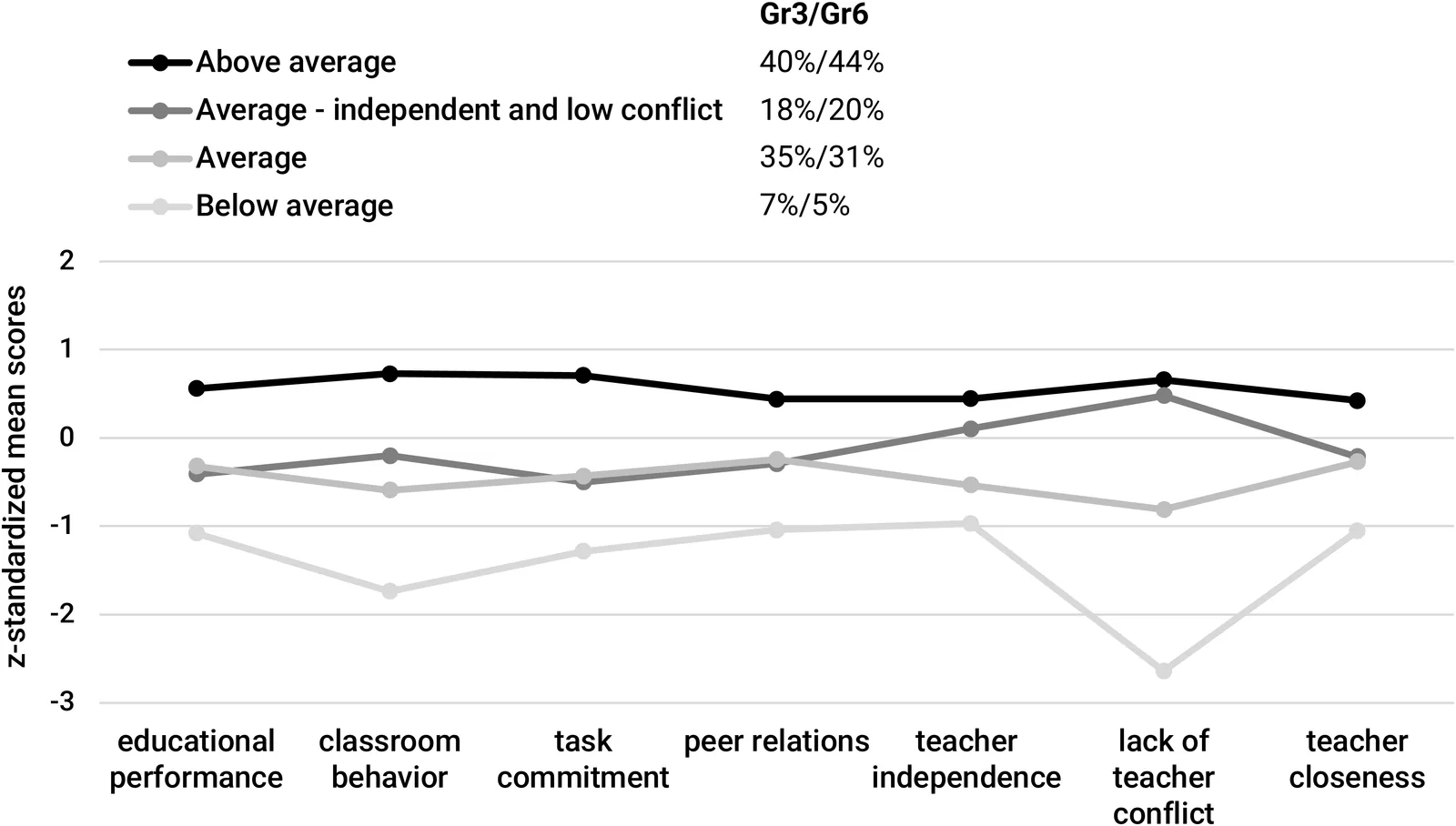
Grade-invariant profiles of student characteristics (mean scores z-standardized for presentation). Profile sizes in Grade 3/Grade 6 are separated by slash.

The resulting solution with time-invariant means can be seen in Table 8 and Figure 2. The four profiles were labeled according to their mean scores on the variables: (1) *above average,* (2) *average – independent and low conflict,* (3) *average,* and (4) *below average*. *Above average* students (39.77–43.99%) formed the largest group and were rated favorably on all student characteristics. *Average* students (30.68–34.90%) were rated moderately on all student characteristics. *Average – independent and low conflict* students (18.77–19.96%) were rated similarly to the *average* students*,* except on lack of teacher conflict and teacher independence, on which they were rated more favorably. *Below average* students (5.37%–7.07%) were rated least favorably on all student characteristics, especially on classroom behavior and lack of teacher conflict.

**Table 8:** Classification of gifted students in grade-invariant profiles of student characteristics based on their most likely latent profile membership.

Student characteristic	Time-invariant means			
	Above average (*n* = 526/581)	Average– independent and low conflict (*n* = 242/264)	Average (*n* = 461/406)	Below average (*n* = 93/71)
Educational performance	3.31	2.53	2.55	1.99
Classroom behavior	4.51	3.81	3.54	2.53
Task commitment	4.49	3.50	3.54	2.85
Peer relations	4.16	3.59	3.55	2.84
Teacher independence	3.59	3.32	2.89	2.45
Lack of teacher conflict	3.96	3.87	2.94	1.61
Teacher closeness	3.93	3.61	3.53	3.07

Profile sizes in Grade 3/Grade 6 are separated by slash.

### 3.2 Stability of profile membership over time

**Figure 3:**
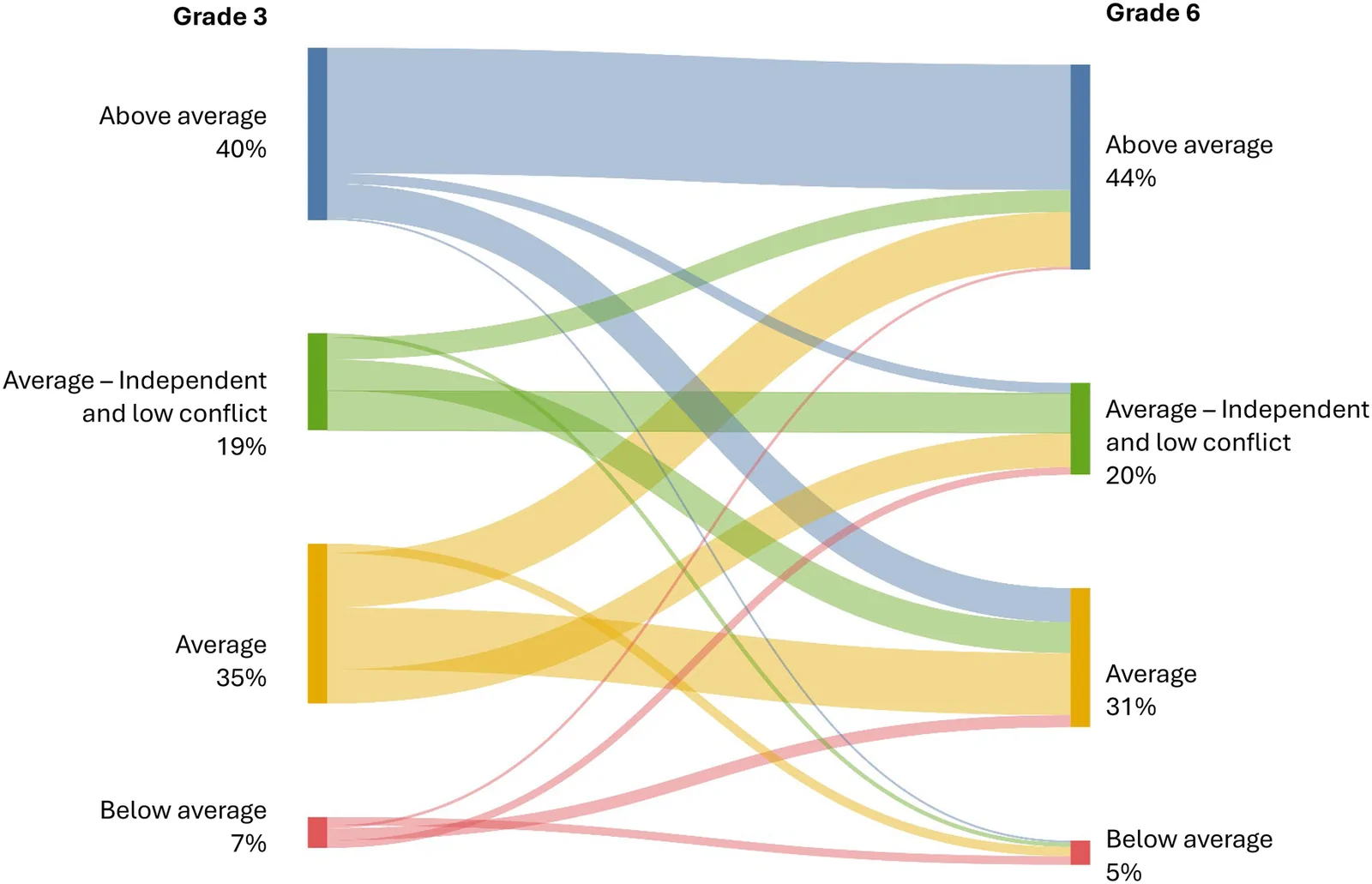
Transitions between profiles of gifted students from Grade 3 to Grade 6. Thickness of lines corresponds with the size of transition probabilities. Straight lines indicate the transition probabilities for classification in the same profiles over time. Diagonal lines mark the transition probabilities for classification in other profiles over time.

Our second aim was to investigate whether differences in profile size could be identified over time, and whether students made transitions between profiles over time. Table 8 suggests that the number of students in each profile did not vary much across both measurement waves. Table 9 shows the results for the LTA i.e., the cross-classification of the profile membership across time and the transition probabilities. The boldface diagonal in Table 9 represents profile stability i.e., the percentage of students who were classified in the same profile over time and the average probability of being classified in the same profile over time. In total, 51% of the sample was classified in the same profile over time. For the 49% of the sample that did change profiles, twelve possible transitions could have been observed. As shown in Table 9 and Figure 3, the transition probabilities indicated that some transitions seemed more likely than others (87). The highest transition probabilities were found for the consistent assignment to the same profile over time, except for the *below average* profile. Transition probabilities confirmed systematic patterns of transitions to other profiles. Likely transitions (chosen criterion: *N* > 30; transition probability >.10) included transitions from less favorable to more favorable profiles, and vice versa. Transitions were likely for *above average* students to *average* students, for *average – independent and low conflict* students to *above average* and *average* students, for *average* students to *above average* and *average – independent and low conflict* students, and for *below average* students to *average* and *average – independent and low conflict* students.

**Table 9:** Cross-classification of profile membership (%), transition probabilities, based on most likely latent class pattern.

Grade 3	Grade 6							
	Above average		Average– independent and low conflict		Average		Below average	
	cross-classification(%)	transition probabiity	cross-classification(%)	transition probabiity	cross-classification(%)	transition probabiity	cross-classification(%)	transition probabiity
Above average	**27**.**33**	(.**70**)	2.30	(.09)	7.45	(.20)	0.44	(.01)
Average– independent and low conflict	4.75	(.28)	**8**.**60**	(.**36**)	6.76	(.32)	0.88	(.04)
Average	11.87	(.33)	7.40	(.23)	**13**.**43**	(.**39**)	2.04	(.06)
Below average	0.62	(.10)	1.69	(.23)	2.63	(.41)	**1**.**82**	(.**26**)

The boldface diagonal represents profile stability i.e., the percentage of students who were classified in the same profile over time and the average probability of being classified in the same profile over time.

### 3.3 Profile membership associations with achievement and achievement growth

**Figure 4:**
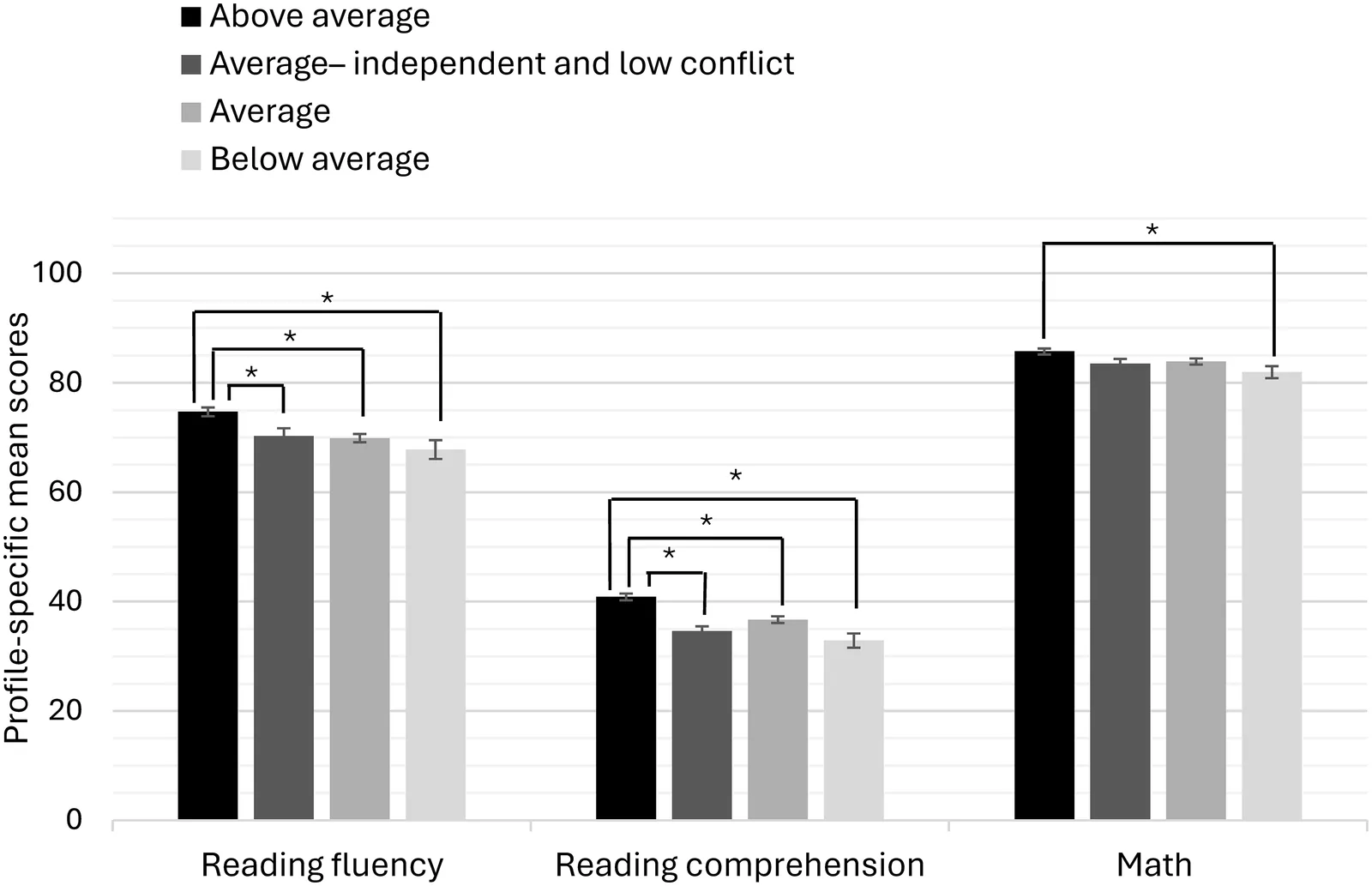
Comparisons between Grade 3 profiles on achievement outcomes in Grade 3. *significant for *α*_SID_ < .0028. Vertical bounded lines indicate standard error.

**Figure 5:**
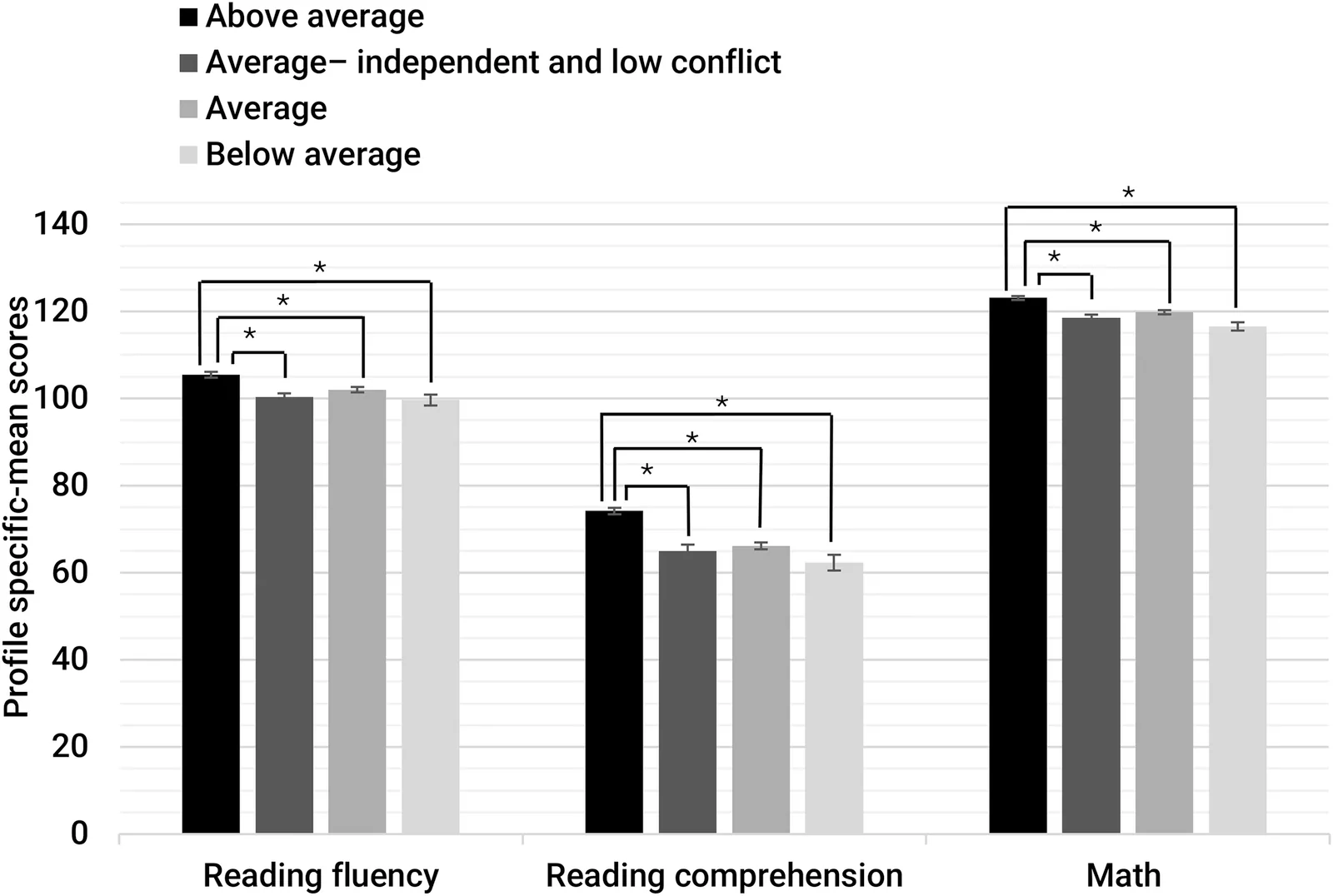
Comparisons between Grade 6 profiles on achievement outcomes in Grade 6. *significant for *α*_SID_ < .0028.

**Figure 6:**
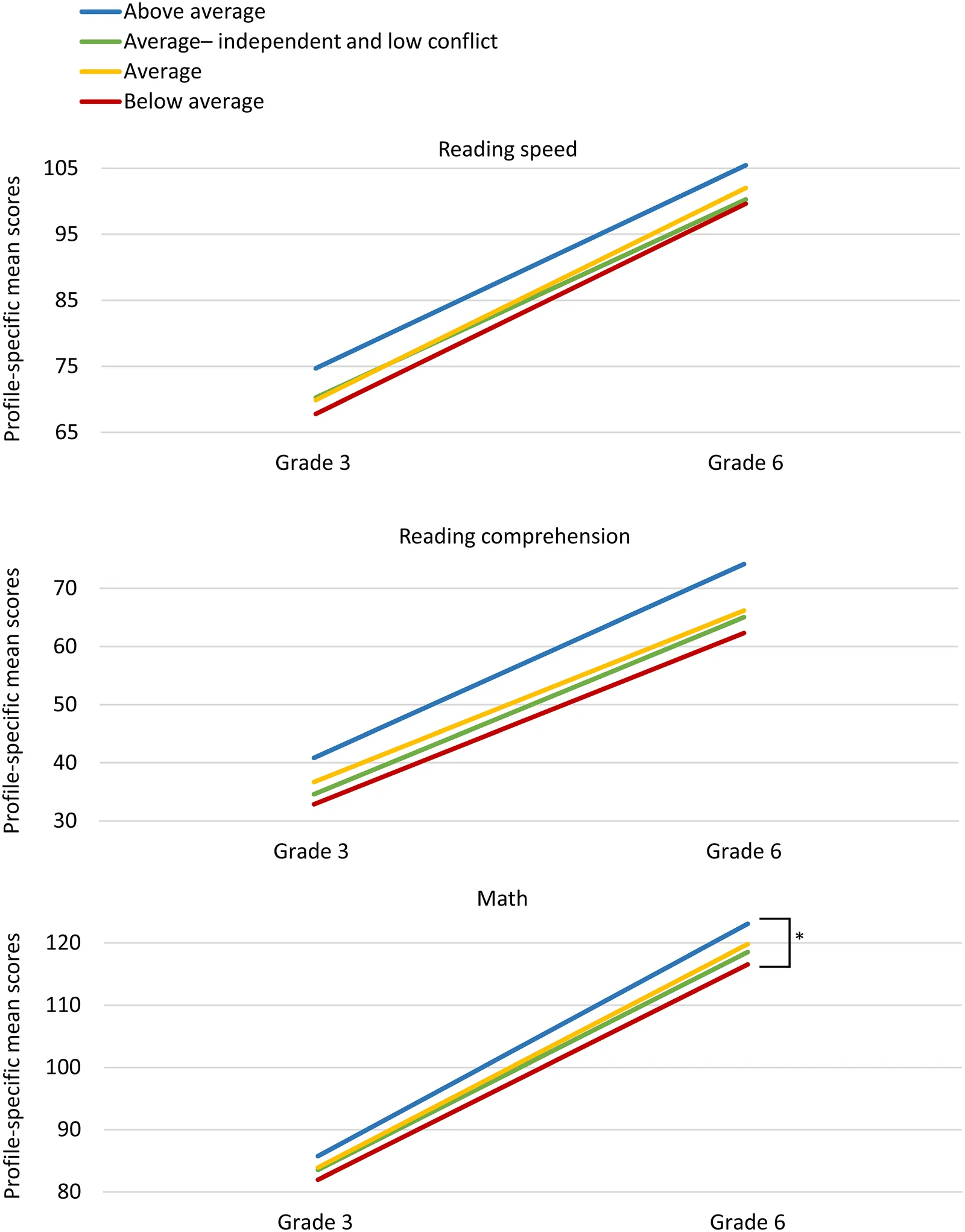
Comparisons between Grade 3 profiles on achievement growth between Grades 3 and 6. *significant for *α*_SID_ < .0028.

Our last aim was to investigate whether students in different profiles differed in achievement and achievement growth. Results of Wald tests comparing the means of the different profiles on the standardized school achievement tests for reading fluency, reading comprehension and math in Grade 3 and 6, are shown in Figures 4–6.

Regarding the question to what extent Grade 3 profiles differed in achievement outcomes in Grade 3, we found that the *above average* profile outperformed all other profiles on reading fluency and reading comprehension (see Figure 4). On math, the *above average* students only outperformed the *below average* students. The effect size of the difference between the *above average* profile and the *below average* profile on reading comprehension was medium (*d* *=* .63), effect sizes of other differences were small (*d_range_ =* .26 -.49). Overall, there were no significant differences between the *average – independent and low conflict*, *average* and *below average* students.

Regarding the question whether Grade 6 profiles are associated with achievement outcomes in Grade 6, we found that the *above average* profile outperformed the other profiles on reading fluency, reading comprehension, and math (see Figure 5). There were no significant differences between the other profiles. Effect sizes on reading comprehension were medium (*d_range_ *= .50 -.74), as well as the difference between the *above average* profile and the *below average profile* on math (*d* *=* .68); other differences were small (*d*_range_* =* .27 -.47).

To compare profiles on differences in their achievement growth, we investigated differences between Grade 3 profiles on their mean test scores in grade 6, while correcting for the scores in Grade 3. On math, we found that the *above average* profile showed significantly higher achievement growth than the *below average* profile (see Figure 6); this was a small effect, *d* *=* .32. This was not found for reading fluency and reading comprehension. Overall, there were no significant differences between the *average – independent and low conflict, average,* and *below average* profiles.

## 4 Discussion

This study aimed to extend previous research on profiles among gifted students by examining whether different profiles could be distinguished among gifted students in primary school, based on student characteristics that are observable by teachers: educational performance, classroom behavior, task commitment, teacher-child relationship quality, and peer relations. Moreover, we investigated to what extent the identified profiles were stable over time and related to achievement and achievement growth. The findings revealed four distinct profiles of gifted students, further supporting previous research (6, 19) showing that gifted students do not constitute a homogeneous group. Adding to previous work, the present study suggests that these quantitatively distinct groups of gifted students can be differentiated based on teacher ratings of students’ characteristics. Even though students across the four profiles were all characterized by high cognitive abilities, they differed in their levels of achievement and, to some extent, their achievement growth. This suggests that, overall, these student characteristics help to explain why some gifted students do not perform at the level predicted by their cognitive abilities, while others with similar abilities achieve highly. These findings suggests that teachers’ observations of both cognitive and non-cognitive characteristics may contribute to the identification of gifted students who may be at risk for underachievement. Importantly, the differences between profiles were primarily quantitative in nature—that is, the profiles varied mainly in the overall levels of the characteristics rather than representing qualitatively different patterns. Moreover, even though similar profiles emerged in Grade 3 and 6, only about half of the students remained in the same profile after three years. This indicates that profile membership of gifted students does not represent a stable, fixed disposition. In the following paragraphs, the outcomes regarding the prevalence, stability, and predictive value of the profiles for achievement growth are discussed in more detail.

### 4.1 The prevalence of profiles among gifted students

A first goal of the present study was to examine which profiles were present among gifted students. Contrary to most previous research, our study focused on teachers’ perceptions to examine whether teachers can identify gifted children at risk of underachievement based on their perceptions of a range of student characteristics. Four profiles of gifted students were distinguished based on a variety of student characteristics as observed by their teachers. These four profiles were labeled (1) *above average*, (2) *average – independent and low conflict*, (3) *average*, and (4) *below average*. The findings are consistent with contemporary research on giftedness that shows that gifted students – like all other children - vary in cognitive and non-cognitive characteristics (19) and are in line with previous profile research suggesting that distinct profiles of gifted students can be distinguished (37, 39, 45, 46). However, the profiles revealed in the current study mostly represented quantitative differences, in contrast to the widespread belief in significant qualitative differences between profiles of gifted students (profiles characterized by specific student characteristics such as *the successful* and *the creative*) (8–11) and previous profile research (27, 28, 35, 37, 38). Similar to our study, some other profile studies among gifted students focusing on a specific range of student characteristics (such as bullying, self-concept, hope, or intrinsic motivation) also found mostly quantitative rather than qualitative differences between profiles (29, 33, 45, 46). In some of these studies, student characteristics were likely to co-occur, for example aspects of self-concept in primary school students (88). In our study, we did not find a high correlation between student characteristics (correlations up to .64). A possible explanation is that teacher ratings of different student characteristics were highly consistent, because teachers have a general positive, moderate or negative view of each student, the ‘halo effect’ (the tendency to overgeneralize the evaluation of an individual's positive or negative qualities (89);. Nonetheless, our findings suggest that some qualitative differences between profiles are observable by teachers. That is, the below average group showed unfavorable teacher ratings in general, but had even worse scores on teacher conflict and classroom behavior. The two average groups also differed in teacher conflict and classroom behavior, as well as in teacher independence, with one group showing better scores on these characteristics. Teacher conflict exhibited the greatest variance.

### 4.2 Stability of profile prevalence and profile membership over time

A second goal of the present study was to examine whether the same profiles and profile sizes occurred after three years and whether students changed profiles over time. As previous research suggests that students’ characteristics change over time (45–48), we expected changes in profile membership over time. Contrary to our expectations, similar profiles were found over time, indicating that similar types of students could be distinguished over longer time periods based on teacher ratings of students’ characteristics. Moreover, profile sizes were highly stable, which suggests that equivalent numbers of students were classified in more and less favorable profiles over time. Half of the sample was classified in the same profile after three years. Among those students who transitioned between profiles, the percentage of students transitioning to more favorable profiles over time was almost equivalent to the percentage of students transitioning to less favorable profiles, with slightly more transitions in positive directions. These findings suggest that profile membership should not be seen as stable, fixed disposition of gifted students. Given that most of the student characteristics included in the profiles are malleable and subject to change over time, some degree of profile transition was to be expected. Moreover, because students in Dutch primary schools typically have different teachers in Grades 3 and 6, changes in teacher perceptions may also have contributed to shifts in profile membership.

### 4.3 Profile membership associations with achievement and achievement growth

A third goal of this study was to examine the predictive value of profile membership for achievement and achievement growth. In both grades, we found differences between the profiles in achievement. Students in the most favorable profiles outperformed students in the other profiles in math, reading fluency, and reading comprehension. Moreover, for math, profile membership predicted achievement growth over time. These findings show that positive task commitment, classroom behavior, relationships with classmates, and the teacher-child relationship are important preconditions for gifted students’ achievement. Moreover, in line with previous research (44), the results suggest that gifted students who score below average on these characteristics, show lower achievement outcomes. Possibly, this points to a self-fulfilling prophecy (90): teacher expectations of students’ educational performance become true through their consequence. Hence, the current study indicates that students with unfavorable scores on student characteristics could be at risk of underachievement. These results suggest that teachers observations of students’ task commitment, classroom behavior, educational performance, peer relations and teacher-child relationship quality may contribute to the identification of gifted students who are at risk of underachievement.

### 4.4 Limitations and suggestions for future research

Some limitations of this study need to be considered when interpreting our results. First, a limitation of this study is the use of data collected in 2007–2014. Since then, regular education classrooms have undergone several changes. For example, technology has become more widely integrated into educational practice. Additionally, and perhaps more relevant to the present study, there has been increasing attention in the Netherlands to meeting the educational needs of gifted students (91, 92). These developments may have influenced how gifted students are taught (for instance, an increasing number of regular schools now offer pull-out programs). However, these changes are not necessarily expected to have altered teachers’ perceptions of gifted students’ characteristics or the associations between different profiles of teacher-perceived characteristics and students’ achievement (growth). Therefore, the main findings of the present study are likely still applicable. Nevertheless, studies using more recent data, if available, could further examine the extent to which these findings hold in the current educational context.

Second, using data from a large existing dataset enabled us to examine a large group of gifted students and include a substantial number of relevant student characteristics, yet this dataset did not include measures of other variables that could also be of interest. For example, creativity could not be included in the current study, although this could also be an important student characteristic in distinguishing different types of gifted students (8, 93).

Third, although the three-year longitudinal design of this study enabled us to investigate changes over longer time periods, students usually change teachers when they are in different grades. Therefore, the moderate stability of profile membership over time could in part be due to differences between teachers in their evaluation of students. That is, the changes in profile membership could partly be due to differences between Grade 3 and Grade 6 teachers in their ratings. With the design of our study, we could not establish to what extent differences were attributable to changes in student characteristics between Grade 3 and 6 or to differences between teachers in how they rated their students. Importantly though, disentangling these sources of change was not the primary aim of the study. Rather, our interest was in understanding how teacher-rated profiles of gifted students evolve over time in an educational context in which students usually have different teachers in different school years. In this respect, the study has strong ecological validity, as the findings reflect the way gifted students are observed and evaluated across different stages of primary education. Longitudinal studies with more frequent measurement waves could explore to what extent profile membership is stable within and across school years, based on teacher ratings from the same teacher.

Fourth, to generalize our results to gifted students in general, this study needs to be replicated with different age groups. Another suggestion for future research is to explore whether the use of other teacher questionnaires can reveal more qualitatively different profiles. Future research could include a more extensive teacher questionnaire (focusing on additional aspects of student characteristics), and/or observational measures for teachers. To gain insight into factors that promote favorable cognitive, social-emotional, behavioral, and motivational characteristics in gifted students, future research could focus on antecedents of profile membership and examine which characteristics of the home and school environment are associated with profile membership. This would provide further insight into factors that can predict trajectories toward underachievement.

## 5 Conclusion

First, this study adds to the body of knowledge showing that gifted students form a heterogeneous group differing in cognitive and non-cognitive student characteristics. However, based on teacher perceptions, gifted students display less divergent student characteristics than assumed in previous research (8). That is, if teachers evaluated students positively on certain characteristics, they were likely to evaluate them positively on other student characteristics too. Nonetheless, this study shows that distinct groups of students can be distinguished based on teacher ratings of students’ characteristics. Second, findings show that these profiles were related to achievement (growth). As such, students’ cognitive and non-cognitive characteristics, as observed by their teachers as early as in Grade 3, could help to identify students at risk for underachievement. Negative task commitment, negative classroom behavior, poor educational performance, and low-quality relationships with classmates and teachers were factors related to gifted students’ underachievement. The finding that profile membership predicted achievement and achievement growth also implies that teachers may help prevent underachievement among gifted students by creating a learning climate that contributes positively to the factors underlying these profiles.

## Data Availability

Publicly available datasets were analyzed in this study. This data can be found here: https://ssh.datastations.nl/dataset.xhtml?persistentId=doi:10.17026/DANS-XTK-UZ6K; Data Archiving and Networked Services (DANS).
